# Environmental Enrichment Improves Cognitive Deficits, AD Hallmarks and Epigenetic Alterations Presented in 5xFAD Mouse Model

**DOI:** 10.3389/fncel.2018.00224

**Published:** 2018-08-15

**Authors:** Christian Griñán-Ferré, Vanesa Izquierdo, Eduard Otero, Dolors Puigoriol-Illamola, Rubén Corpas, Coral Sanfeliu, Daniel Ortuño-Sahagún, Mercè Pallàs

**Affiliations:** ^1^Department of Pharmacology and Therapeutic Chemistry, Institut de Neurociències, University of Barcelona, Barcelona, Spain; ^2^Institut d’Investigacions Biomèdiques de Barcelona (IIBB), CSIC, IDIBAPS and CIBERESP, Barcelona, Spain; ^3^Laboratorio de Neuroinmunomodulación Molecular, Instituto de Investigación en Ciencias Biomédicas (IICB), Centro Universitario de Ciencias de las Salud (CUCS), Universidad de Guadalajara, Guadalajara, Mexico

**Keywords:** behavior, cognition, environmental enrichment, epigenetics, APP, Tau, oxidative stress, inflammation

## Abstract

Cumulative evidence shows that modifications in lifestyle factors constitute an effective strategy to modulate molecular events related to neurodegenerative diseases, confirming the relevant role of epigenetics. Accordingly, Environmental Enrichment (EE) represents an approach to ameliorate cognitive decline and neuroprotection in Alzheimer’s disease (AD). AD is characterized by specific neuropathological hallmarks, such as β-amyloid plaques and Neurofibrillary Tangles, which severely affect the areas of the brain responsible for learning and memory. We evaluated EE neuroprotective influence on 5xFAD mice. We found a better cognitive performance on EE vs. Control (Ct) 5xFAD mice, until being similar to Wild-Type (Wt) mice group. Neurodegenerative markers as β-CTF and tau hyperphosphorylation, reduced protein levels whiles APPα, postsynaptic density 95 (PSD95) and synaptophysin (SYN) protein levels increased protein levels in the hippocampus of 5xFAD-EE mice group. Furthermore, a reduction in gene expression of *Il-6, Gfap, Hmox1 and Aox1* was determined. However, no changes were found in the gene expression of neurotrophins, such as Brain-derived neurotrophic factor (*Bdnf*), Nerve growth factor (*Ngf*), Tumor growth factor *(Tgf)* and Nerve growth factor inducible (*Vgf*) in mice with EE. Specifically, we found a reduced DNA-methylation level (5-mC) and an increased hydroxymethylation level (5-hmC), as well as an increased histone H3 and H4 acetylation level. Likewise, we found changes in the hippocampal gene expression of some chromatin-modifying enzyme, such as *Dnmt3a/b*, *Hdac1*, and *Tet2*. Extensive molecular analysis revealed a correlation between neuronal function and changes in epigenetic marks after EE that explain the cognitive improvement in 5xFAD.

## Introduction

Emerging evidence indicates that the aberrant transcriptional regulation of memory-related genes reflects epigenetic landscape modifications because of changes in environmental factors (Puckett and Lubin, 2011; Duncan et al., 2014; Maloney and Lahiri, 2016). On the other hand, the main driving force of senescence process is the differential regulation of gene expression by epigenetic mechanisms (López-Otín et al., 2013). Moreover, a distinctive and critical influence of diet, Environmental Enrichment (EE) or exercise have emerged as strategies that correlate preventing cognitive decline and improving quality of life (Ricci et al., 2018).

Alzheimer disease (AD) and other dementias are strongly associated with aging (Delgado-Morales et al., 2017). AD involves degeneration of certain regions of the brain, which results in memory loss and declining in cognition, functions and behavior. The neuropathological hallmarks comprise the accumulation and deposition of β-amyloid in the senile plaques, and hyperphosphorylated tau (p-Tau) protein, a microtubule assembly protein formation of Neurofibrillary Tangles (NFTs; Bloom, 2014). Additionally, there are other key pathological changes such as oxidative stress, neuroinflammation and neuronal loss (Agostinho et al., 2010).

Epigenetic could act as the primary mechanism contributing to the pathogenesis of AD, with a critical role in the interaction between the genome and the environment (Spiegel et al., 2014; McCreary and Metz, 2016). In particular, modifications in DNA methylation (5-mC) and hydroxymethylation (5-hmC) are seen in the brain tissues of AD mouse model (Chouliaras et al., 2010; Lunnon et al., 2014; Griñán-Ferré et al., 2016c). Furthermore, deregulation of histone acetylation has been implicated in various neurodegenerative disorders such as AD (Bahari-Javan et al., 2012). Likewise, alterations in microRNA (miRNA) expression were found in the hippocampus of both demented patients (Lau et al., 2013) and AD mouse models (Barak et al., 2013; Cosín-Tomás et al., 2014). Specifically, dysregulation in *miR-101* (Long and Lahiri, 2011), *miR-153* (Long et al., 2012) and *miR-339-5* (Long et al., 2014), downregulates the APP expression in mouse *in vitro* and *in vivo* models and AD patients. In fact, several miRNAs have been proposed as biomarkers for AD (Yılmaz et al., 2016).

EE is an excellent experimental paradigm able to induce oxidative stress reduction (Pusic et al., 2016), a curbing in inflammation (Jurgens and Johnson, 2012) and epigenetic changes (Irier et al., 2014). EE is based on housing conditions that provide a combination of social interactions, cognitive, sensory and motor stimulation (Rosenzweig and Bennett, 1996; van Praag et al., 2000; Mora et al., 2007). In fact, it has been widely described the influence of the environment on behavior and cognition (Nathianantharajah and Hannan, 2006). For instance, a number of studies have demonstrated that rodent AD models maintained under EE conditions show better cognitive performance correlated with beneficial changes in the brain (Griñán-Ferré et al., 2016a,b; Hüttenrauch et al., 2016). Nevertheless, the mechanisms by which EE alters brain structure and function are not well understood. Since Hebb’s first EE experiments, the two main mechanisms described were that EE promoted changes at the anatomical and electrophysiological level (Irvine et al., 2006; Eckert and Abraham, 2010). An alternative mechanism was that EE promoted neural plasticity through increasing levels of growth factors such as brain-derived neurotrophic factor (BDNF) and nerve growth factor (NGF), among others, in the brain (Ickes et al., 2000; Angelucci et al., 2009). There is also evidence that EE attenuates both oxidative stress (Herring et al., 2010; Cechetti et al., 2012) and inflammatory process (McQuaid et al., 2013).

Recent studies have demonstrated that EE promotes changes in DNA methylation states at the global level or at specific loci while changing the expression of DNA MethylTransferases (DNMTs; Madrigano et al., 2011; Barrès et al., 2012; Griñán-Ferré et al., 2016b). Other global changes in histone acetylation H3/H4 have been seen in AD mouse model (Fischer et al., 2007; Griñán-Ferré et al., 2016c; Vierci et al., 2016). Besides, it has been reported changes in DNA methylation of *Bdnf* promoter in rat hippocampus, caused increases in gene expression after EE (Gomez-Pinilla et al., 2011).

5xFAD represents an important transgenic murine model of AD, which develops early and aggressive hallmarks of amyloid burden and cognitive loss (Oakley et al., 2006; Devi and Ohno, 2010; Girard et al., 2013). Additional AD pathologies exhibited by the 5xFAD model include age-dependent synaptic degeneration (Wang et al., 2016), mitochondrial dysfunction (Devi and Ohno, 2012), increase in oxidative stress (Griñán-Ferré et al., 2016c), and microglial activation (Landel et al., 2014). On the other hand, epigenetic alterations in the 5xFAD model were also described (Anderson et al., 2015). Remarkably, recent studies revealed a correlation among cognitive deficits, Aβ pathology and epigenetic alterations (Griñán-Ferré et al., 2016c), demonstrating the key role of epigenetics in this mouse model.

The present work aimed to confirm the association between EE and cognitive improvement in 5xFAD mice and the molecular changes observed. Besides, we went deep to highlight the putative correlation between epigenetic alterations and the mechanisms underlying the neurodegenerative process in AD.

## Materials and Methods

### Animals and Housing Conditions

Female Wild-Type (Wt-Ct, *n* = 24) and 5xFAD (*n* = 24) mice were used to carry out cognitive. Animals had free access to food and water and were kept under standard temperature conditions (22 ± 2°C) and 12 h:12 h light-dark cycles (300 lux/0 lux).

**Figure 1:**
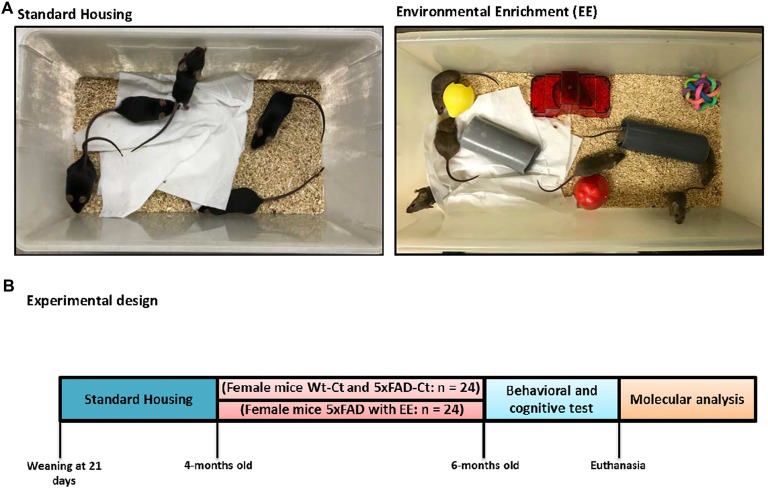
Housing conditions and experimental design. Exemplary pictures of standard (Control, Ct) and enriched (Environmental Enrichment, EE) housing conditions **(A)**. Mice were housed in groups of 5–6. Enriched cages were equipped with tunnels, houses, and toys. At 4-month-old 5xFAD were randomly allocated to either Ct or EE conditions for 8 weeks. With 6-month-old, mice were tested in behavioral and memory tests followed by euthanasia and tissue collection **(B)**.

The animals were maintained until 4-month-old with standard conditions, and afterward were separated into treatment groups at up to 6-month-old. Twelve Wt and 5xFAD were employed for the EE group during 8 weeks (Wt-EE and 5xFAD-EE), and 12 were maintained under standard conditions as Control mice (Wt-Ct and 5xFAD-Ct). In the present study, we utilized the novel objects paradigm to accomplish EE conditions. Therefore, plastic tubes (20 cm long and 2.5 cm in diameter) were placed in EE cages, in addition to plastic dolls or toys, which were added, extracted, or changed each week (Figure 1).

This study was carried out in accordance with the recommendations of European Community Council Directive 86/609/EEC and the procedures established by the Department d’Agricultura, Ramaderia i Pesca of the Generalitat de Catalunya, Spain. Every effort was made to minimize animal suffering and to reduce the number of animals.

### Behavioral Tests

#### Elevated Plus Maze

The Elevated Plus Maze (EPM) was performed as previously described (Griñán-Ferré et al., 2016a). Forty-eight mice (*n* = 12 per group) were placed on the central platform, facing an open arm, and allowed to explore the apparatus for 5 min. After the 5-min test, each mouse was returned to their home cages, and the EPM apparatus was cleaned with 70% ethyl alcohol and allowed to dry between tests. Behavior was scored with SMART ver. 3.0 software and each trial were recorded for later analysis. Parameters recorded included time spent on open arms, time spent on closed arms, time spent in the center zone, rearings, defecation and urination.

#### Open Field Test

The Open Field Test (OFT) was performed as previously described (Griñán-Ferré et al., 2016a). Forty-eight mice (*n* = 12 per group) were individually placed at the center and allowed to explore the white polywood box (50 × 50 × 25 cm) for 5 min. Behavior was scored with SMART^®^ ver.3.0 software and each trial was recorded for later analysis. The parameters scored included center-staying duration, rearings, defecations and the distance traveled.

#### Novel Object Recognition Test

The Novel Object Recognition Test (NORT) protocol employed was a modification of Ennaceur and Delacour (1988) and Ennaceur and Meliani (1992). In brief, 48 mice (*n* = 12 per group) were placed in a 90°, two-arm, 25-cm-long, 20-cm-high, 5-cm-wide black maze. Before performing the test, mice were individually habituated to the apparatus for 10 min during 3 days. On day 4, the animals were submitted to a 10-min acquisition trial (first trial), during which they were placed in the maze in the presence of two identical, novel objects at the end of each arm. After a delay (2 h and 24 h), the animal was exposed to two objects one old object and one novel object. The time that mice explored the Novel object (TN) and Time that mice explored the Old object (TO) were measured. A Discrimination Index (DI) was defined as (TN − TO)/(TN + TO). In order to avoid object preference biases, objects were counterbalanced.

#### T-Maze Spontaneous Alternation

T-Maze spontaneous alternation from 48 samples (*n* = 12 per group) was tested as previously described (Fragkouli et al., 2005). The protocol we followed consisted of one forced and 10 consecutive free-choice trials on the same day. Briefly, on the first trial, mice were individually placed on the central stem of the T-maze allowing to explore the central stem and one of the goal arms of the maze. After entering the goal arm, exit from the arm was blocked, and mice were left to explore the arm for 30 s. Animals were then retrieved, and the testing cycle started again 10 min later with another free-choice trial. The percentage of alternation (number of turns in each goal arm) and total trial duration are recorded and calculated.

### Immunodetection Experiments

#### Brain Processing

Mice were euthanized by cervical dislocation one day after the behavioral test finished. Brains were immediately removed from the skull. The hippocampus was then isolated and frozen in powdered dry ice. They were maintained at −80°C for further use. Tissue samples were homogenized in lysis buffer containing phosphatase and protease inhibitors (Cocktail II, Sigma). Total protein levels were obtained and protein concentration was determined by the method of Bradford.

#### Protein Levels Determination by Western Blotting

For Western Blotting (WB), aliquots of 15 μg of hippocampal protein were used. Protein samples from 12 samples (*n* = 4 per group) were separated by SDS-PAGE (8%–12%) and transferred onto PVDF membranes (Millipore). Afterwards, membranes were blocked in 5% non-fat milk in 0.1% Tween20 TBS (TBS-T) for 1 h at room temperature, followed by overnight incubation at 4°C with the primary antibodies listed in Supplementary Table S1.

Afterward, membranes were washed and incubated with secondary antibodies for 1 h at room temperature. Immunoreactive proteins were viewed with a chemiluminescence-based detection kit, following the manufacturer’s protocol (ECL Kit; Millipore) and digital images were acquired using a ChemiDoc XRS+ System (BioRad). Semi-quantitative analyses were carried out using ImageLab software (BioRad) and results were expressed in Arbitrary Units (AU), considering control protein levels as 100%. Immunodetection of GAPDH, or β-Actin routinely monitored protein loading.

#### Determination of Oxidative Stress in Hippocampus

Hydrogen peroxide from 12 samples (*n* = 4 per group) was measured as an indicator of oxidative stress, and it was quantified using the Hydrogen Peroxide Assay Kit (Sigma-Aldrich, St. Louis, MI) according to the manufacturer’s instructions.

#### Global DNA Methylation and Hydroxymethylation Quantification

Isolation of genomic DNA from 12 samples (*n* = 4 per group) was conducted using the FitAmpTM Blood and Cultured Cell DNA Extraction Kit according to the manufacturer’s instructions. Then, Methylflash Methylated DNA Quantification Kit (Epigentek, Farmingdale, NY, USA) and MethylFlash HydroxyMethylated DNA Quantification Kit were used in order to detect methylated and hydroxymethylated DNA. Briefly, these kits are based on specific antibody detection of 5-mC and 5-hmC residues, which trigger an ELISA-like reaction that allows colorimetric quantification by reading absorbance at 450 nm using a Microplate Photometer.

#### Global Histone Acetylation H3 and H4 Quantification

Histone extracts from 12 samples (*n* = 4 per group) were prepared by using a total histone extraction kit (Epigentek) according to the manufacturer’s protocol. Detection of global histone H3/H4 acetylation status was performed using the EpiQuik™ global histone H3/H4 acetylation assay kit (Cat. P-4008-96/P-4009-96, Epigentek Group Inc., NY, USA), following the manufacturer’s recommendations. Briefly, histone proteins (1–2 μg) were added to the strip wells. Acetylated histone H3/H4 was detected with a high-affinity antibody, and the ratios and amounts of acetylated histone H3/H4 were displayed with a horseradish peroxidase-conjugated secondary anti-body color development system. The color was measured by reading absorbance at 450 nm using a Microplate Photometer.

#### RNA Extraction and Gene Expression Determination

Total RNA isolation from 12 samples (*n* = 4 per group) was carried out by means of TRIzol^®^ reagent following the manufacturer’s instructions. The yield, purity, and quality of RNA were determined spectrophotometrically with a NanoDrop™ ND-1000 (Thermo Scientific) apparatus and an Agilent 2100B Bioanalyzer (Agilent Technologies). RNAs with 260/280 ratios and RIN higher than 1.9 and 7.5, respectively, were selected. Reverse Transcription-Polymerase Chain Reaction (RT-PCR) was performed as follows: 2 μg of messenger RNA (mRNA) was reverse-transcribed using the High Capacity cDNA Reverse Transcription Kit (Applied Biosystems). Real-time quantitative PCR (qPCR) was employed to quantify the mRNA expression of a set of chromatin-modifying enzyme genes, oxidative stress genes, inflammatory genes, neurotrophin genes. Normalization of expression levels was performed with actin for SYBER Green and TATA-binding protein (*Tbp*) for TaqMan. Primers and TaqMan probes are listed in Supplementary Table S2.

SYBR^®^ Green real-time PCR was performed in a Step One Plus Detection System (Applied-Biosystems) employing SYBR^®^ Green PCR Master Mix (Applied-Biosystems). Each reaction mixture contained 7.5 μL of complementary DNA (cDNA; which concentration was 2 μg), 0.75 μL of each primer (which concentration was 100 nM), and 7.5 μL of SYBR^®^ Green PCR Master Mix (2×).

TaqMan-based real-time PCR (Applied Biosystems) was also performed in a Step One Plus Detection System (Applied-Biosystems). Each 20 μL of TaqMan reaction contained 9 μL of cDNA (25 ng), 1 μL 20× probe of TaqMan Gene Expression Assays and 10 μL of 2× TaqMan Universal PCR Master Mix.

Data were analyzed utilizing the comparative Cycle threshold (Ct) method (ΔΔCt), where the housekeeping gene level was used to normalize differences in sample loading and preparation. Normalization of expression levels was performed with *actin* for SYBR^®^ Green-based real-time PCR results and TATA-binding protein *(Tbp)* for TaqMan-based real-time PCR. Each sample was analyzed in duplicate, and the results represent the n-fold difference of the transcript levels among different groups.

#### Data Analysis

The statistical analysis was conducted using GraphPad Prism ver. 6 statistical software. Data are expressed as the mean ± Standard Error of the Mean (SEM) of at least four samples per group. Means were compared with one-way analysis of variance (ANOVA), followed by Tukey *post hoc* test. Statistical significance was considered when a *p*-values were <0.05. The statistical outliers were determined with Grubbs’ test and subsequently removed from the analysis.

## Results

### Effect of EE, in Locomotor Activity and Motivational in 5xFAD Mice

**Figure 2:**
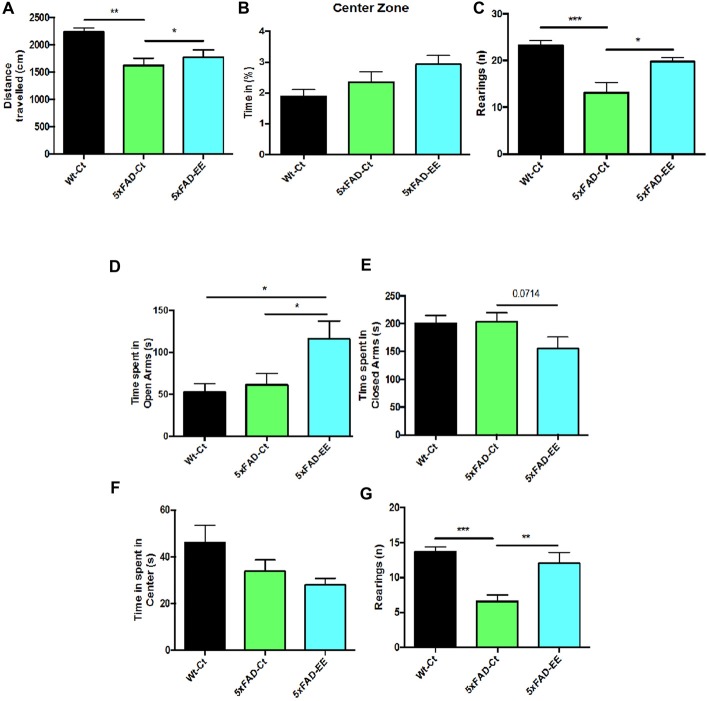
Results of Open Field Test (OFT) in female mice at 4-month-old wild-type (Wt)-Ct and 5xFAD-Ct mice groups and 5xFAD mice group after 8 weeks with EE. Distance traveled **(A)**, percentage of time spent in Center zone **(B)** and Rearings **(C)**. Results of Elevated Plus Maze (EPM) in female mice at 4-month-old Wt-Ct and 5xFAD-Ct mice groups and 5xFAD mice group after 8 weeks with EE. Time spent in Open Arms **(D)**. Time spent in Closed Arms **(E)**. Time spent in Center **(F)** and Rearings **(G)**. Values represented are mean ± Standard error of the mean (SEM); *p < 0.05; **p < 0.01; ***p < 0.001.

5xFAD-EE group restored the locomotor activity in comparison with Wt-Ct group (Figure 2A). Time spent in the center zone was higher in 5xFAD-EE, although did not reach significance (Figure 2B) as compared with controls. Furthermore, a significant increase in vertical activity, quantified by the number of rearings, in comparison with the 5xFAD-Ct was found (Figure 2C). Results obtained in the OFT exhibited changes in fear behavior and motor activity. Additional parameters measured in the EPM are presented in Supplementary Table S3.

The specific anxiety value obtained in the EPM, time spent in opened arms, showed a significant increase in 5xFAD-EE compared to 5xFAD-Ct (Figure 2D); time spent in closed arms by 5xFAD-EE mice group was slightly lower than other groups (Figure 2E). Furthermore, time spent in the center was lower in 5xFAD-EE, although did not reach significance (Figure 2F) as compared with controls. Finally, accordingly with OFT results, a significant increase in 5xFAD-EE vertical activity compared to 5xFAD-Ct was found (Figure 2G). Results obtained in the EPM demonstrated changes in fear-anxiety-like behavior. Additional parameters measured in the EPM are presented in Supplementary Table S4.

### EE Reduced Cognitive Deficits Presented by 5xFAD Mice

**Figure 3:**
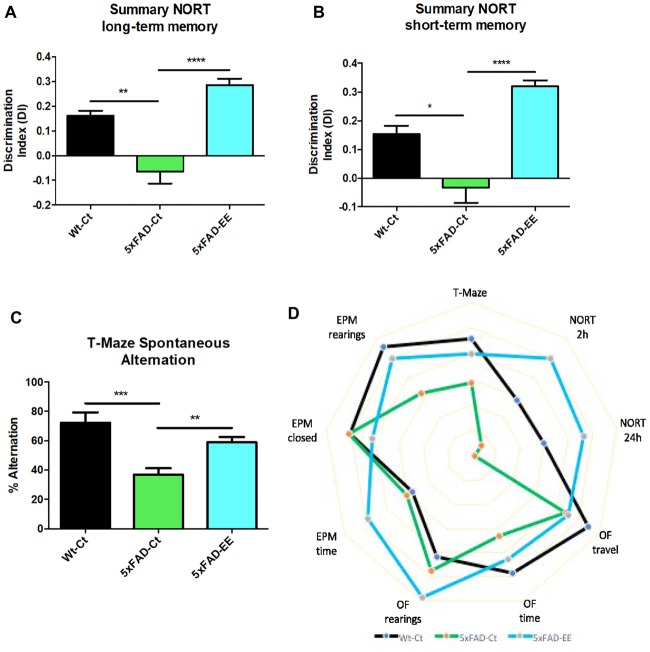
Results of Discrimination Index (DI) of Novel Object Recognition Test (NORT) in female mice at 4-month-old Wt-Ct and 5xFAD-Ct mice groups and 5xFAD mice group after 8 weeks with EE. Summary from short-term memory **(A)**, and from summary long-term memory **(B)**. Results of Spontaneous Alternation T-Maze in female mice at 4-month-old Wt-Ct and 5xFAD-Ct mice groups and 5xFAD mice group after 8 weeks with EE. Percentage successful spontaneous alternations in a T-Maze **(C)**. Polygonal graph presented complete parameters obtained by OF, EPM, NORT and T-Maze **(D)**. Values represented are mean ± Standard error of the mean (SEM); *p < 0.05; **p < 0.01; ***p < 0.001; ****p < 0.0001.

NORT analysis showed, as described (Griñán-Ferré et al., 2016c) an impaired short- and long-memory in 5xFAD mice in comparison with age mated Wt (Supplementary Figure S1). However, EE intervention did not modify memory capabilities in Wt mice (Supplementary Figure S1). By contrast, NORT analysis demonstrated that 5xFAD-EE mice exhibited significantly reduced cognitive deficits in both short- and long-term memory (Figures 3A,B), obtaining highest DI than 5xFAD-Ct. Moreover, we found significant differences in spontaneous alternation between Wt-Ct and 5xFAD-Ct (Figure 3C) and between 5xFAD-EE and 5xFAD-Ct (Figure 3C), but not for Wt-EE (Supplementary Figure S1). The absence of changes in cognitive parameters in Wt mice, conducted us to evaluate only epigenetic marks in this strain (Supplementary Figure S2).

Finally, the polygonal graph depicts differences between 5xFAD mice with EE and control mice group by graphing several OF parameters, EPM parameters, DI of NORT and spontaneous alternation of T-Maze (Figure 3D), demonstrating that EE intervention improved cognitive performance in 5xFAD mouse model in comparison with Control, approaching it to Wt.

### Hippocampal Global Changes in DNA Methylation and Hydroxymethylation and Its Machinery After EE

**Figure 4:**
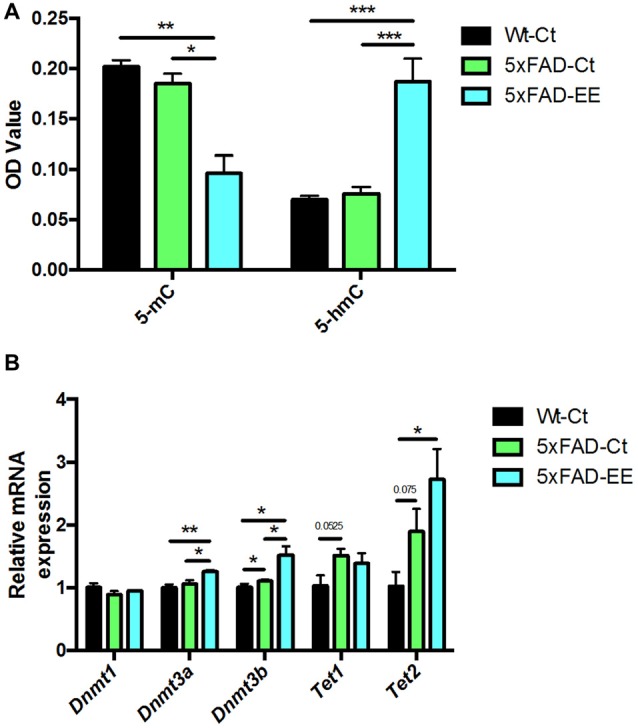
Global 5-methylated and 5-hydroxymethylated cytosine levels in hippocampus **(A)**. Methyltransferases and Hydroxylases gene expression for *Dnmt1*, *Dnmt3a, Dnmt3b, Tet1* and *Tet2* **(B)**. Gene expression levels were determined by real-time PCR. Mean ± Standard Values are mean (SEM) from five independent experiments performed in duplicate are represented; *p < 0.05; **p < 0.01; ***p < 0.001.

To depict a landscape on global methylation affectation by EE we evaluate methylation and hydroxymethylation. 5-methylcytosine levels were significantly reduced in 5xFAD-EE in comparison with control mice (Figure 4A). In parallel, 5-hmC levels were increased in 5xFAD-EE. Because changes in methylation were determined, DNMTs family and Translocation family (TETs), members of DNA hydroxylase family gene expression, were studied; significant increases in *Dnmt3a* and *Dnmt3b* were found in the 5xFAD-EE mice group (Figure 4B). Results showed significant increases in *Tet2*, but not in *Tet1* in the 5xFAD-EE mice group in comparison with control group (Figure 4B). Similar changes in methylation and hydroxymethylation were also determined in Wt-EE mice in comparison with Wt-Ct, accompanied by variations in gene expression levels in enzymes (Supplementary Figure S2).

### Hippocampal Global Changes in Histone Acetylation Levels and Chromatin-Modifying Enzymes After EE

**Figure 5:**
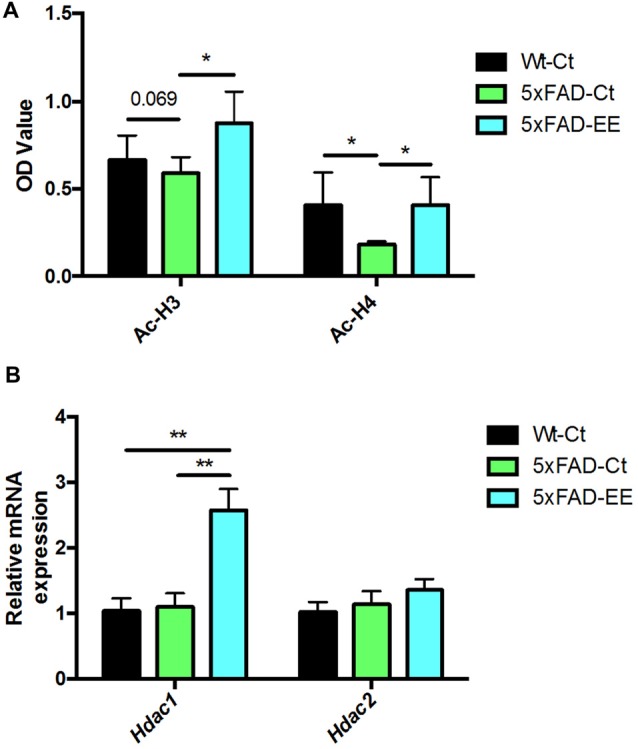
Global histone H3/H4 acetylation levels in hippocampus **(A)**. Deacetylases gene expression related to memory for *Hdac1* and *Hdac2* **(B)**. For Gene expression levels were determined by real-time PCR. Mean ± Standard error of the mean (SEM) from five independent experiments performed in duplicate are represented; *p < 0.05; **p < 0.01.

Histone acetylation is another epigenetic marker that can modify gene expression. Acetylated H3 and H4 protein levels were significantly increased in 5xFAD-EE compared to 5xFAD-Ct (Figure 5A). Increased gene expression in *Hdac1* but no changes of *Hdac2* were found in 5xFAD-EE in comparison with control mice group (Figure 5B).

### Reduction in Oxidative Stress and Inflammation Markers in 5xFAD Mice

**Figure 6:**
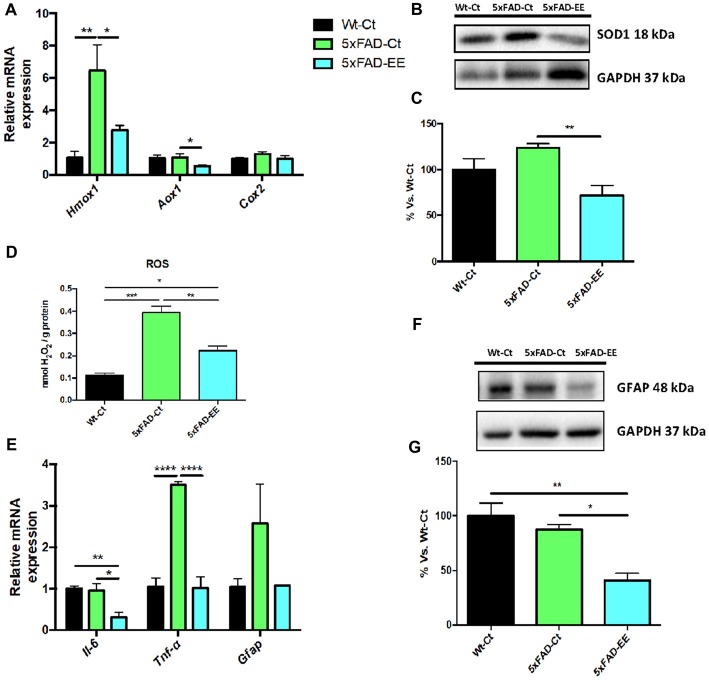
Representative gene expression of antioxidant enzymes for *Hmox1*, *Aox1*, *Cox2* **(A)**. Representative Western blot for SOD1 protein levels **(B)** and quantification **(C)**. Representative Oxidative stress measured as hydrogen peroxide concentration in homogenates of hippocampus tissue **(D)**. Proinflammatory markers *Il-6*, *Tnf-α* and *Gfap* gene expression **(E)**. GFAP protein levels **(F)** and quantification **(G)**. Mean ± Standard error of the mean (SEM) from five independent experiments performed in duplicate are represented; *p < 0.05; **p < 0.01; ***p < 0.001; ****p < 0.0001.

Increases in oxidative stress as well as neuroinflammation are hallmarks of AD. Beneficial effects of EE depicted on a significant reduction in *Hmox1* and *Aox1* between 5xFAD-EE group compared to 5xFAD-Ct (Figure 6A). Likewise, a significant increase in *Hmox1* gene expression (Figure 6A) and SOD1 protein levels (Figures 6B,C) between 5xFAD-Ct in comparison with Wt-Ct was found. Moreover, a slight but not significant decrease in *Cox2* gene expression in the 5xFAD-EE was observed when compared to the control group (Figure 6A). Finally, analysis of hydrogen peroxide levels in homogenates of hippocampus tissue showed a significant decrease in ROS levels in 5xFAD-EE compared to 5xFAD-Ct (Figure 6D). Interestingly, the highest ROS levels were observed in 5xFAD-Ct.

On the other hand, EE induced a significant reduction in *Il-6* and *Tnf-α* gene expression compared to control groups (Figure 6E). Decreased GFAP protein levels were found in 5xFAD-EE compared to control groups (Figures 6F,G). In addition, Wt-Ct and 5xFAD-EE presented a slight, although not significant, diminution in *Gfap* gene expression compared to 5xFAD-Ct (Figure 6E).

### Increased Synaptic Marker Protein Levels but Not in Neurotrophins Gene Expression After EE

**Figure 7:**
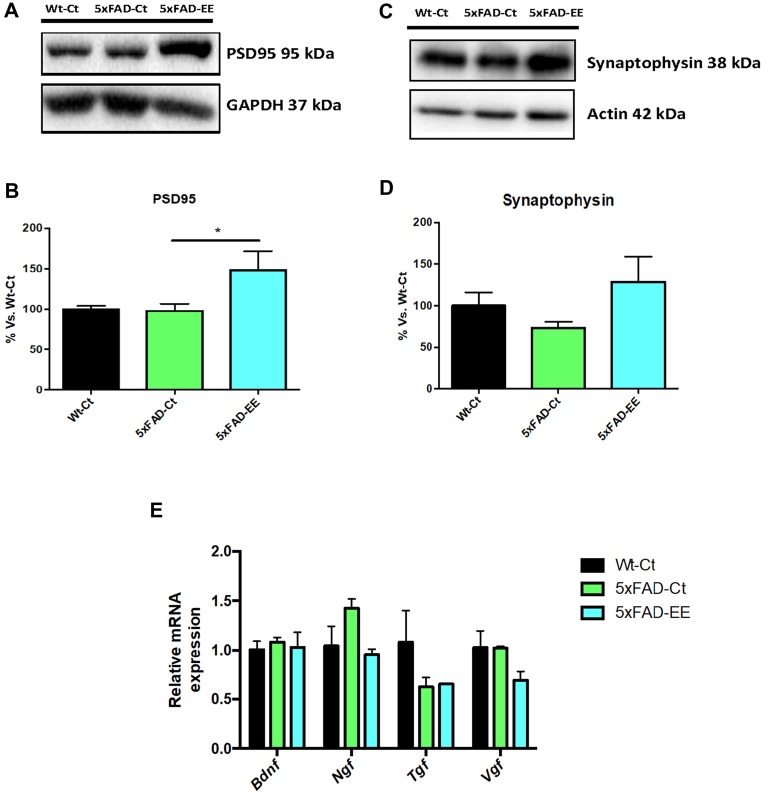
Neuroplasticity markers in female mice at 4-month-old Wt-Ct and 5xFAD-Ct mice groups and 5xFAD mice group after 8 weeks with EE. Representative Western blot for postsynaptic density 95 (PSD95) protein levels protein levels **(A)** and quantification **(B)**, synaptophysin (SYN; **C**) and quantification **(D)**. Values in bar graphs are adjusted to 100% for protein levels of control (Wt-Ct). Representative gene expression for *Brain-derived neurotrophic factor (BDNF), Nerve growth factor (NGF), Tgf* and *Vgf* **(E)**. Gene expression levels were determined by real-time PCR. Values are mean ± Standard error of the mean (SEM); *p < 0.05.

Synaptic damage and neurotrophin expression alteration have been described in 5xFAD. A significant increase in postsynaptic density 95 (PSD95) protein levels was found in 5xFAD-EE compared to 5xFAD-Ct reaching Wt-Ct levels (Figures 7A,B). Albeit did not reach significance, EE increased synaptophysin (SYN) protein levels (Figures 7C,D). However, no significant differences were observed in *Bdnf*, *Ngf*, *Tgf*, and *Vgf* gene expression after EE or among 5xFAD and control mice groups (Figure 7E).

### EE Changed Alzheimer’s Disease Markers in 5xFAD Mice

**Figure 8:**
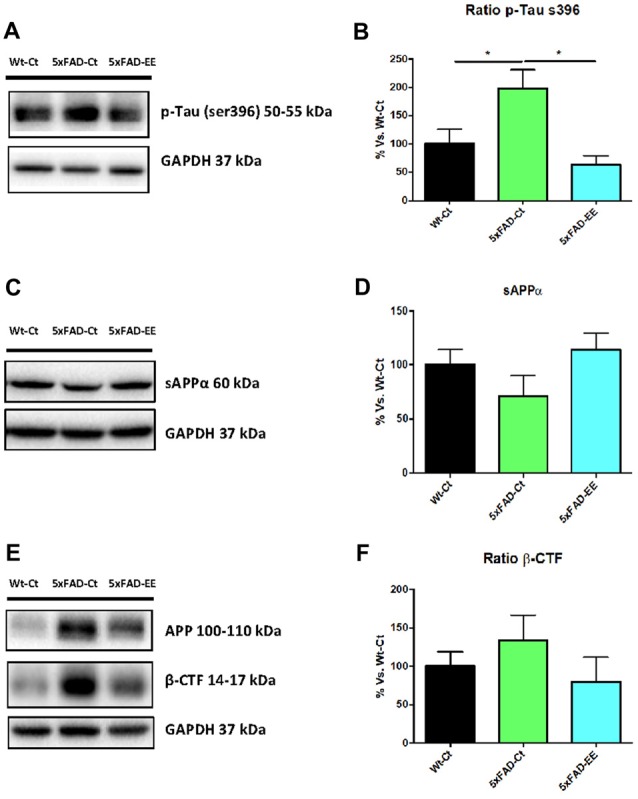
Alzheimer’s disease markers in female mice at 4-month-old Wt-Ct and 5xFAD-Ct mice groups and 5xFAD mice group after 8 weeks with EE. Representative Western blot for pTau ser396 protein levels **(A)** and quantifications **(B)**, protein levels of sAPPα and β-CTF **(C,E)** and quantifications **(D,F)**. Values in bar graphs are adjusted to 100% for protein levels of control Wild-Type (Wt-Ct); *p < 0.05.

As mentioned, 5xFAD mice is an established AD model with changes in amyloid cascade and tauopathy. Prevention by EE of these two AD hallmarks was studied. To this end, we evaluate tau hyperphosphorylation and APP processing in 5xFAD under EE. Western blot analysis revealed that EE produced a significant decrease in both p-Tau Ser396 in 5xFAD reaching WT-Ct levels (Figures 8A,B). Additionally, a tendency to increase in sAPPα protein levels was found in 5xFAD-EE reaching Wt-Ct levels (Figures 8C,D), whereas β-CTF (the fragment delivered by β-secretase) was diminished after EE intervention (Figures 8E,F).

## Discussion

The present study aimed to evaluate, whether an EE intervention is effective in 5xFAD mouse model, modifying behavioral, cognitive and molecular AD hallmarks present in this transgenic mouse model. To this end, changes in oxidative stress, inflammation, tau hyperphosphorylation, and APP processing markers were determined. Besides, some epigenetic markers were studied to elucidate a possible epigenetic mechanism linking EE, molecular changes and better cognition status. The enrichment was started at the age of 4-month-old, when cognitive deficit appear in 5xFAD mice (Girard et al., 2013), and was continued until the age of 6-month-old.

Alterations in epigenetics contribute to gene expression deregulation, resulting in the development of different pathologies in mouse models and human beings, including unhealthy aging and AD (Hook et al., 2018; Griñán-Ferré et al., 2018). In previous reports, changes in cognition and molecular pathways were found after exercise or EE in a mouse model for senescence and early-AD. Modification of those parameters related to neurodegeneration was correlated with epigenetic modifications (Cosín-Tomás et al., 2014; Griñán-Ferré et al., 2016b). There are a number of works describing epigenetic alterations induced by EE and controlling several molecular pathways such as neuroplasticity and neuroinflammation (Williamson et al., 2012; Yang et al., 2012).

Our group described epigenetic modifications in 5xFAD (Griñán-Ferré et al., 2016c) and demonstrated that behavioral, biochemical and molecular changes in this AD mice model correlated with epigenetic changes. Although many studies related to beneficial effects of EE have been described in 5xFAD mice (Hüttenrauch et al., 2016; Ziegler-Waldkirch et al., 2018), this is, to our knowledge, the first time that EE has been connected to epigenetic mechanisms intervention with beneficial effects on cognition in 5xFAD mice.

EE for 8 weeks was enough to induce better cognitive performance in 5xFAD, including an improvement in spatial and recognition memory (Griñán-Ferré et al., 2016b; Hüttenrauch et al., 2016), in concordance to other works in different transgenic strain and with exercise (García-Mesa et al., 2016), that is other enrichment paradigm. In fact, the action of EE depends on the type of EE, experimental design with different duration of exposure, the severity of AD mouse model phenotype, age, and gender, among others. Besides, behavioral task analysis showed more active and less anxiety-like behavior gated to 5xFAD mice under EE.

As mentioned above, 5-mC is a stable epigenetic mark that alters the neuronal function to promote changes in gene expression through environment interactions such as behavior, stress, hormones, and EE that can participate in memory function (Keil and Lein, 2016). DNA methylation and hydroxymethylation constitute epigenetic markers that can alter neuronal function influencing gene expression. Accordingly, cognitive and behavioral changes after EE in Wt and 5xFAD were reflected in epigenetic marks 5-mC and 5-hmC changes.

Recent data suggest an aberrant 5-mC formation is linked to neurodegeneration and apoptotic neuronal death (Chestnut et al., 2011; Hernandez et al., 2011). Likewise, altered expression levels and pathological activity DNMT have been observed in aging and neurodegenerative disorders (Johnson et al., 2012). After EE, 5-mC levels were reduced in 5xFAD, but, conversely, *Dnmt3a/b* gene expressions were increased, whereas *Dnmt1* gene expression was not modified. In fact, DNA methylation changes by DNMT activity are necessary for memory formation and storage (Day and Sweatt, 2010). For instance, it was reported that elimination of DNMT1 in mice brain causes a hypomethylation in cortical and hippocampal neurons, resulting in the neurodegenerative process with deficits learning and memory (Hutnick et al., 2009). In addition, Oliveira et al. (2012) found in aged mice that increased gene expression of *Dnmt3a2* was associated with cognitive decline prevention, accordingly with results obtained in 5xFAD-EE.

5-hmC, an oxidative product of DNA demethylation is another epigenetic mark catalyzed by the TET family that exists at high levels within the brain (Irier et al., 2014). In our study, EE exposure increased 5-hmC levels and *Tet2* gene expression in 5xFAD mice. Indeed, these results are consistent with those reported recently by Irier et al. (2014) in mice. Besides, it has been described that loss of *TET2* induces DNA hypermethylation in a similar way to oxidative stress, reducing global 5-hmC (Zhang et al., 2017). In contrast, no changes in *Tet1* expression were found after EE, although in other experimental study were described (Irier et al., 2014); different timing and duration of the EE and also for the strain and age can explain differences.

Histone acetylation is one of the epigenetic modifications, which play a vital role in the etiopathogenesis of AD (Lu et al., 2015). Overall, HDAC inhibition increases histone acetylation H3/H4 and rescued learning and memory deficits in AD animal models, showing neuroprotective effects (Lu et al., 2015; Neidl et al., 2016). Here, we indeed reported a significant increase in H3 and H4 acetylation levels after EE in 5xFAD. Besides, the participation of histone acetylases and HDACs in AD is controversial. For example, it has been reported that HDAC2, but not HDAC1 or HDAC3, is up-regulated in AD mouse models (Broide et al., 2007), but other reported differential changes in HDAC family levels, for example, the SAMP8 mouse model (Griñán-Ferré et al., 2016b). In contrast with this last report, an increase of *Hdac1* expression in the 5xFAD-EE whereas no changes in *Hdac2* expression were found.

Thus, it can be hypothesized that epigenetic changes induced by EE should lead to a relaxed chromatin structure, allowing gene expression increase and protein synthesis essential for learning and memory (Kim and Kaang, 2017). These results suggest the role of EE in epigenetic modifications restoring learning and memory in an AD mouse model.

Besides cognitive and behavioral improvement induced by EE in 5xFAD mice, molecular and biochemical pathways related to AD pathogenic mechanism had been studied. Oxidative stress is accepted to have a key role in Aβ-induced neurotoxicity (Cheignon et al., 2018). The oxidative stress occurs when the balance between antioxidant enzymes and ROS are disrupted (Birben et al., 2012; Poljsak et al., 2013). Up-regulation of antioxidant enzymes might confer protection against oxidative insults (Chen et al., 2011). Specifically, changes in SOD1 expression and HMOX1 (Sugawara et al., 2002; Guo et al., 2010) were found, in response to a wide variety of oxidative stress stimuli. EE reduced H_2_O_2_ levels indicating oxidative stress diminution, and accompanied by lower SOD1 protein levels. Moreover, we observed a reduction in gene expression of the antioxidant enzymes *Hmox1* and *Aox1*.

Likewise, there is evidence that neuroinflammation plays a role in aging and the pathophysiology of AD (Liu et al., 2015; Raj et al., 2017). Significant differences in proinflammatory chemokines secreted by astrocytes have been proved between AD patients and normal aged people (Liu et al., 2015). A reduction in gene expression for *Il-6* and *Tnf-α* in 5xFAD-EE mice suggested a reduction in the inflammatory process in 5xFAD (Gurel et al., 2018). The lower inflammation after EE was also reinforced by a diminution in astroglial activation after EE demonstrated a significant decrease in *Gfap* gene expression and protein levels (Brahmachari et al., 2006). Evidence concerning food containing natural antioxidant and anti-inflammatory compounds open the avenue to potentiate a lifestyle based on diet, exercise and a healthy environment to underlying neurophysiological mechanisms in AD or neurodegenerative diseases, as a non-pharmacological strategy to face the cognitive impairment (Businaro et al., 2018; Francis and Stevenson, 2018).

As aforementioned, EE induced experience-dependent synaptic plasticity (Nithianantharajah et al., 2004), increased synapse and spine density (Jung and Herms, 2014) and memory consolidation through increased expression of synaptic proteins (Hu et al., 2010) and growth factors (Ickes et al., 2000). Accordingly, several reports in which an EE intervention showed improvements on hippocampal-dependent task (Jurgens and Johnson, 2012; Stein et al., 2016; Cortese et al., 2018) increased levels of both proteins PSD95 (postsynaptic) and SYN (presynaptic) in the hippocampus were found in 5xFAD-EE, indicating a neuroprotective role of EE intervention.

In our study, we were unable to determine any effect on gene expression of neurotrophic factors (NTFs) such as *Bdnf, Ngf*, *Tgf* and *Vgf* in 5xFAD with EE. Previous reports highlighted the influence of EE on molecular mediators of synaptic plasticity in AD mice models is inconsistent. While several studies found an increase in NTFs (Tong et al., 2001; Angelucci et al., 2009; Jha et al., 2016), others did not found differences in some of them (Hüttenrauch et al., 2016). There are several NTFs that are involved in neuroprotective effects of EE, and it is probable that experimental conditions such as type and duration of EE modifies growing factors. By last, about AD hallmarks, EE diminished p-Tau (Ser396) in the hippocampus of the 5xFAD.

In conclusion, our results confirm previous data were supporting the beneficial effects of EE in AD mouse models. Concretely, improvements in cognitive performance associated with changes in oxidative stress, inflammation, synaptic plasticity, and AD hallmarks were found in 5xFAD mice. Remarkably, those changes paralleled with epigenetic mechanisms in 5xFAD, as described previously in the non-transgenic model of cognitive impairment and senescence (Griñán-Ferré et al., 2016a,b), disentangling a link between epigenetics, lifestyle and neurodegeneration.

It has been proposed that epigenetic mechanisms can modify the onset, latency period and progression of neurodegenerative diseases and this work give support to this claim that has emerged in the last decade (Tsankova et al., 2007). Strengthening the mechanistic understanding of neurodegeneration and its correlation with epigenetics, as is demonstrated in the 5xFAD model, will likely provide new insights pointing out the importance for the healthy lifestyle in the individuals at risk for AD. Results obtained in animal models must be validated in human beings but, noteworthy confirmation for epigenetic alterations in AD patients (Narayan and Dragunow, 2017) and the goodness of an improved lifestyle (diet, exercise, etc; Chouliaras et al., 2010; Ricci et al., 2018) is day by day more robust, evidencing the interrelation between neurodegeneration and epigenetics.

## Author Contributions

CG-F, EO and DP-I carried out the experimental intervention. CG-F and EO performed behavior experiments. RC and DP-I performed Western blot analysis. CG-F and VI performed the RT-PCR experiments. CG-F, DO-S and CS analyzed the data and drafted the manuscript. MP designed the experiments and supervised the study. All authors read and approved the final manuscript.

## Conflict of Interest Statement

The authors declare that the research was conducted in the absence of any commercial or financial relationships that could be construed as a potential conflict of interest.
