# Supplementary material for: Environmental Enrichment Improves Cognitive Deficits, AD Hallmarks and Epigenetic Alterations Presented in 5xFAD Mouse Model
Source: Front Cell Neurosci. 2018 Aug 15;12:224. doi: 10.3389/fncel.2018.00224 (PMC6104164; doi:10.3389/fncel.2018.00224)
Supplement: Supplementary file 1 [file Table_1.DOCX]

**Table 1.** Antibodies used in Western blot studies.

| Antibody | Host | Source/Catalog | WB dilution |
| --- | --- | --- | --- |
| GFAP | Rabbit | Abcam/ab48050 | 1:1000 |
| SOD1 | Sheep | Calbiochem/574597 | 1:1000 |
| Synaptophysin | Rabbit | Dako/CloneSY38 | 1:2000 |
| PSD95 | Rabbit | Abcam/ab18258 | 1:1000 |
| APP C-Terminal | Mouse | Covance/SIG-39152 | 1:1000 |
| sAPPα | Rabbit | Covance/SIG-39139 | 1:1000 |
| p-Tau Ser396 | Rabbit | Invitrogen/44752G | 1:1000 |
| p-Tau Ser404 | Rabbit | Invitrogen/44758G | 1:1000 |
| Tau total | Goat | Santa Cruz/sc-1995 | 1:1000 |
| Actin | Mouse | Sigma-Aldrich/A5441 | 1:2000 |
| GAPDH | Mouse | Millipore/MAB374 | 1:5000 |
| Goat-anti-mouse HRP  conjugated |  | Biorad/170-5047 | 1:2000 |
| Donkey-anti-goat HRP  conjugated |  | Santa Cruz/sc-2020 | 1:2000 |
| Rabbit-anti-sheep HRP conjugated |  | Abcam/ab97130 | 1:2000 |
