# Supplementary material for: Environmental Enrichment Improves Cognitive Deficits, AD Hallmarks and Epigenetic Alterations Presented in 5xFAD Mouse Model
Source: Front Cell Neurosci. 2018 Aug 15;12:224. doi: 10.3389/fncel.2018.00224 (PMC6104164; doi:10.3389/fncel.2018.00224)
Supplement: Supplementary file 2 [file Table_2.DOCX]

**Table 2.** Primers and probes used in qPCR studies.

SYBR Green primers

| Target | Product size (bp) | Forward primer (5’-3’) | Reverse primer (5’-3’) |
| --- | --- | --- | --- |
| *Dnmt1* | 85 | ACCTGGAGAGCAGAAATGGC | TGAAAGGGTGTCACTGTCCG |
| *Dnmt3b* | 142 | TGCCAGACCTTGGAAACCTC | GCTGGCACCCTCTTCTTCAT |
| *Tet1* | 188 | CTGCCAACTACCCCAAACTCA | TCGGGGTTTTGTCTTCCGTT |
| *Tet2* | 113 | CCATCATGTTGTGGGACGGA | ATTCTGAGAACAGCGACGGT |
| *Hdac1* | 150 | TCACCGAATCCGCATGACTC | TCTGGGCGAATAGAACGCAG |
| *Hdac2* | 280 | CTATCCCGCTCTGTGCCCT | GAGGCTTCATGGGATGACCC |
| *Aox1* | 286 | CATAGGCGGCCAGGAACATT | TCCTCGTTCCAGAATGCAGC |
| *Cox2* | 126 | TGACCCCCAAGGCTCAAATA | CCCAGGTCCTCGCTTATGATC |
| *Il-6* | 189 | ATCCAGTTGCCTTCTTGGGACTGA | TAAGCCTCCGACTTGTGAAGTGGT |
| *Tnf-α* | 157 | TCGGGGTGATCGGTCCCCAA | TGGTTTGCTACGACGTGGGCT |
| *Gfap* | 125 | CCTTCTGACACGGATTTGGT | ACATCGAGATCGCCACCTAC |
| *Bdnf* | 231 | AGCTGAGCTGTGTGACAGT | TCCATAGTAAGGGCCCGAAC |
| *Ngf* | 111 | GGAGCGCATCGAGTGACTT | CCTCACTGCGGCCAGTATAG |
| *Tgf* | 204 | CAGGGTGAAGGGGAAAACTC | AGTTCGGTCATTCAGTCTCGC |
| *Vgf* | 178 | GTCAGACCCATAGCCTCCC | CTCGGACTGAAATCTCGAAGTTC |
| *Actin* | 190 | CAACGAGCGGTTCCGAT | GCCACAGGTTCCATACCCA |

Taqman probes

| Target | | Product size (bp) | Reference |
| --- | --- | --- | --- |
| *Dnmt3a* | 58 | | Mm00432881_m1 |
| *Hmox1* | 69 | | Mm00516005_m1 |
| *Tbp* | 93 | | Mm00446971_m1 |
