# Supplementary material for: Environmental Enrichment Improves Cognitive Deficits, AD Hallmarks and Epigenetic Alterations Presented in 5xFAD Mouse Model
Source: Front Cell Neurosci. 2018 Aug 15;12:224. doi: 10.3389/fncel.2018.00224 (PMC6104164; doi:10.3389/fncel.2018.00224)
Supplement: Supplementary file 3 [file Table_3.DOCX]

Table 3. Parameters measured in the Open Field Test (OFT). (n): number of events. Results are expressed as a mean ± Standard error of the mean (SEM). **p* <0.05; ***p* <0.01; ****p*<0.001; *****p* <0.0001 vs 5xFAD-Ct. ^#^*p* <0.05; ^##^*p* <0.01; ^###^*p* <0.001; ^####^*p* <0.0001 vs Wt-Ct.

|  | Wt-Ct | 5xFAD-Ct | 5xFAD-EE |
| --- | --- | --- | --- |
| Locomotor activity (cm) | 2,240.22 ± 70.58** | 1,704.10 ± 115.18 | 1,776.98 ± 134.43* |
| Distance in zone-Center (cm) | 51.79 ± 6.10 | 35.68 ± 5.41 | 51.31 ± 5.98 |
| Distance in zone-Periphery (com) | 2,188.43 ± 69.21** | 1,590.30 ± 131.76 | 1,725.67 ± 132.50* |
| Rearings (n) | 23.18 ± 1.13*** | 13.14 ± 2.19 | 19.75 ± 0.94* |
| Grommings (n) | 2.18 ± 0.12 | 4.86 ± 0.40^###^ | 4.75 ± 0.41^###^ |
| Defecations (n) | 0.45 ± 0.21 | 1.00 ± 0.28 | 1.00 ± 0.35 |
| Urinations (n) | 0.00 ± 0.00 | 0.29 ± 0.16 | 0.08 ± 0.08 |
