# Supplementary material for: Environmental Enrichment Improves Cognitive Deficits, AD Hallmarks and Epigenetic Alterations Presented in 5xFAD Mouse Model
Source: Front Cell Neurosci. 2018 Aug 15;12:224. doi: 10.3389/fncel.2018.00224 (PMC6104164; doi:10.3389/fncel.2018.00224)
Supplement: Supplementary file 4 [file Table_4.pdf]

Table 4. Parameters measured in the Elevated Plus Maze Test (EPM). (n): number of events. Results are expressed as a mean ± Standard error of the mean (SEM). *p <0.05; **p <0.01; ***p<0.001; ****p <0.0001 vs 5xFAD-Ct. #p <0.05; ##p <0.01; ###p <0.001; ####p <0.0001 vs Wt-Ct.

	Wt-Ct	5xFAD-Ct	5xFAD-EE
**Total Distance (cm)**	1,098.28 ± 44.89	1,014.43 ± 137.47****	1,131.26 ± 90.23****
**Time in Zone Center (sec)**	45.96 ± 7.53	33.78 ± 4.88	27.92 ± 9.87
**Time in Open Arms (sec)**	53.29 ± 9.47#	61.36 ± 13.66	116.18 ± 21.51*, #
**Time in Closed Arms (sec)**	200.68 ± 13.93	204.13 ± 15.81	155.19 ± 21.12
**Rearings (n)**	13.67 ± 0.70***	6.57 ± 0.95	12.00 ± 1.60**
**Grommings (n)**	4.75 ± 0.37	2.93 ± 0.52	3.69 ± 0.43
**Defecations (n)**	0.00 ± 0.00	0.50 ± 0.20	0.31 ± 0.17
**Urinations (n)**	0.67 ± 0.22	0.64 ± 0.44	0.23 ± 0.23
