# Supplementary material for: Environmental Enrichment Improves Cognitive Deficits, AD Hallmarks and Epigenetic Alterations Presented in 5xFAD Mouse Model
Source: Front Cell Neurosci. 2018 Aug 15;12:224. doi: 10.3389/fncel.2018.00224 (PMC6104164; doi:10.3389/fncel.2018.00224)
Supplement: Supplementary file 5 [file Data_Sheet_1.pdf]

Supplementary material

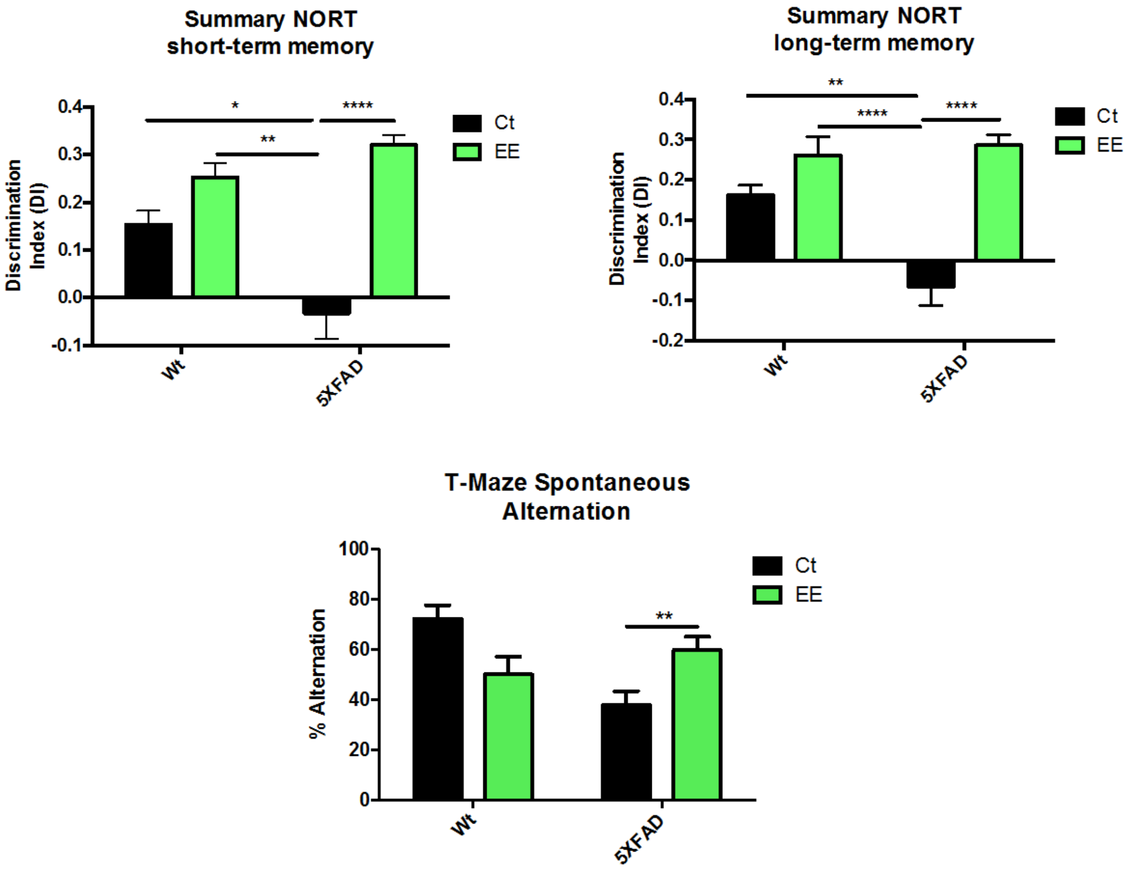

Supplementary Figure 1.

Analysis of short- and long-term memory, and T-maze spontaneous alternation in Wt and 5xFAD animals. Results showed that EE intervention did not influence the cognition capabilities in Wt. Values represented are mean ± Standard error of the mean (SEM); *$p<0.05$; ***p*<0.01; *****p*<0.0001.

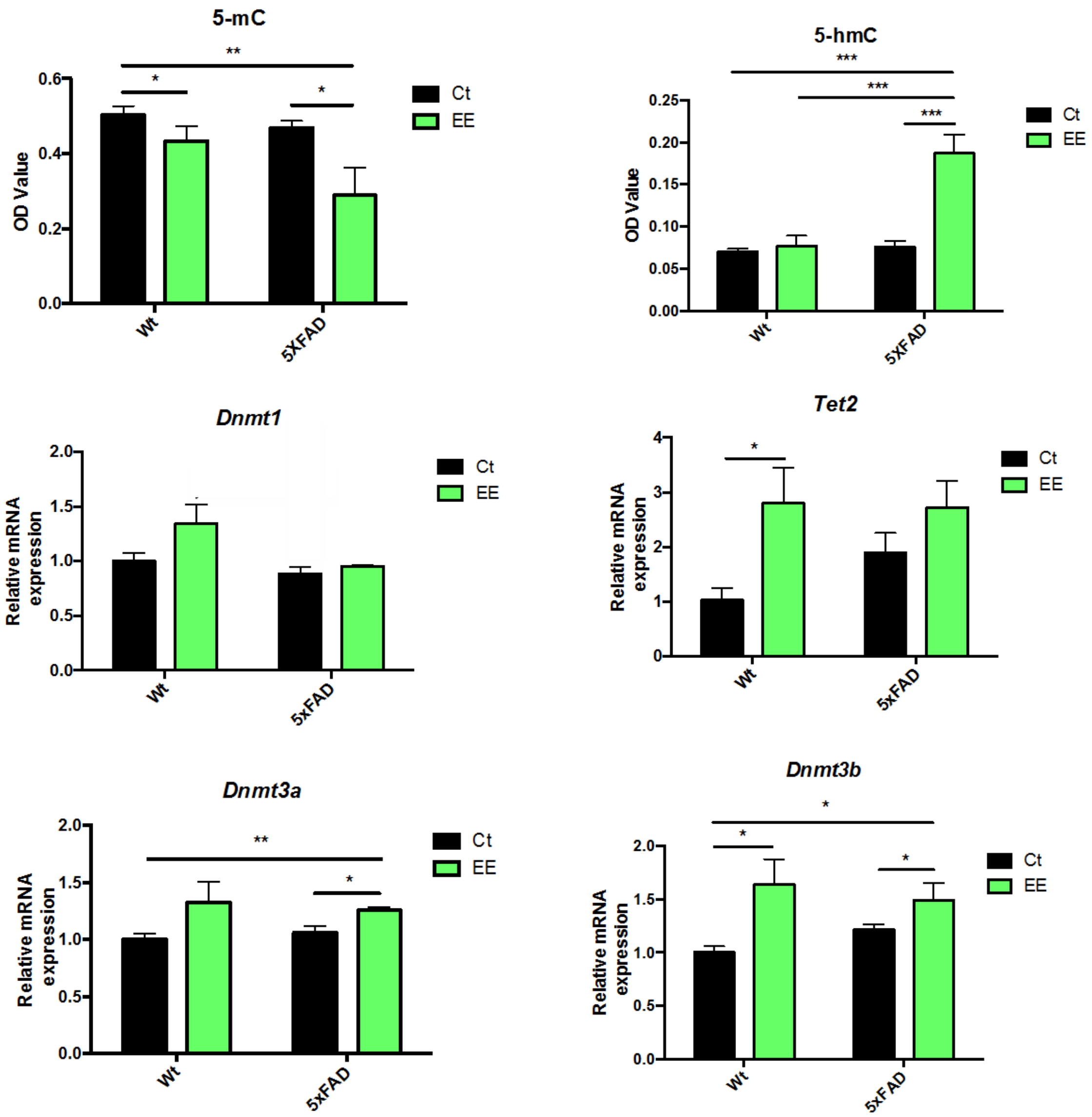

Supplementary Figure 2.

Analysis epigenetic marks. Results showed that EE intervention induced changes in Wt and in transgenic mice in methylation and hydroxymethylation. Several significant changes in enzymes related with those processes have been observed. Values represented are mean ± Standard error of the mean (SEM); *p<0.05; **p<0.01; ***p<0.001.
